# Negligible Long-Term Impact of Nonlinear Growth Dynamics on Heterogeneity in Models of Cancer Cell Populations

**DOI:** 10.1007/s11538-024-01395-w

**Published:** 2025-01-03

**Authors:** Stefano Giaimo, Saumil Shah, Michael Raatz, Arne Traulsen

**Affiliations:** Department of Theoretical Biology, Max Planck Institute for Evolutionary Biology, August-Thienemann-Str. 2, Plön, 24306 Germany

**Keywords:** Cancer, Compartments, Heterogeneity, Linear models, Nonlinear growth

## Abstract

Linear compartmental models are often employed to capture the change in cell type composition of cancer cell populations. Yet, these populations usually grow in a nonlinear fashion. This begs the question of how linear compartmental models can successfully describe the dynamics of cell types. Here, we propose a general modeling framework with a nonlinear part capturing growth dynamics and a linear part capturing cell type transitions. We prove that dynamics in this general model are asymptotically equivalent to those governed only by its linear part under a wide range of assumptions for nonlinear growth.

## Introduction

Populations of cancer cells are typically heterogeneous (Kleppe and Levine 2014; Alizadeh et al. 2015; Pe’er et al. 2021), which means that different types of cells can be identified within the same population on the basis of their different genetic or phenotypic make-ups. Heterogeneity can fuel tumor evolution by affording adaptability, since subpopulations with distinct phenotypes can thrive in distinct somatic environments (Ramón y Cajal et al. 2020) and, in any one of such environments, adaptive peaks may be only a few mutational steps away from some of these subpopulations. Hence, devising sensible targets for tumor therapies is complex and tumors can find ways to evade immune surveillance and drug interventions (McGranahan and Swanton 2017). Reaching a solid understanding of tumor heterogeneity is thus believed pivotal to advance tumor biology and clinical research (Alizadeh et al. 2015; Pillai et al. 2023).

Heterogeneity of cancer cell populations is not a static phenomenon, as cells can change their type, for example via stochastic switching (Gupta et al. 2011). To keep track of the dynamics of different cell types, compartmental models have been employed to model populations of cancer cells. Different compartments correspond to different cell types with transitions between compartments describing the flow of cells through types (Derényi and Szöllősi 2017; Koziol et al. 2020; Magni et al. 2006; Michelson and Leith 1993; Michor et al. 2005; Panetta 1997; Panetta et al. 2006; Simeoni et al. 2004; Werner et al. 2013). Linearity is a common assumption in compartmental models of cancer cells (Panetta and Adam 1995; Panetta 1997; Kozusko et al. 2001; Panetta et al. 2006; Ledzewicz and Schättler 2007; Fornari et al. 2014; Zhou et al. 2014, 2019; Raatz et al. 2021; Li and Thirumalai 2022). In particular, per-capita growth rates of, and transition rates between, cell types, especially prior to treatment, are often supposed to be fixed and independent of population abundances. This assumption can simplify, among other things, the computation of the stationary distribution of cell types (Werner et al. 2013), the sensitivity analysis of growth rates to model parameters (Zhou et al. 2019) and, more in general, the characterization of demographics (e.g. cell age distribution) in the population (Boettcher et al. 2018). From a modeling perspective, such simplification is welcome as model analysis is typically complicated by nonlinearities introduced once treatment regimes are modeled.

But the assumption of linearity implies a population growing exponentially, which is only sustainable for a short time unless model parameters are tuned to guarantee zero growth (Dingli et al. 2007). More realistically, growth dynamics of the total population size of untreated cancer cells as observed in experiments are often described as nonlinear (Benzekry et al. 2014). For example, the formula for logistic growth, which is quadratic in population size (Nowak 2006, Ch. 2), is a usual candidate for fitting growth data of cancer cell populations and a benchmark or an inspiration for alternative, more sophisticated models of these same data (Jin et al. 2017; Bobadilla et al. 2019; Johnson et al. 2019; Sahoo et al. 2021; Roy et al. 2022).

Overall, there is a potential tension between the assumption of linearity, which is convenient for modeling heterogeneity of any sort in populations of cancer cells, and the observation that these populations generally grow in a nonlinear fashion. However, this tension is alleviated by data about non-genetic heterogeneity driven by stochastic switching among cell phenotypes. Experiments in different systems showed that linear models (e.g., Markov chains) accurately predict the dynamics of the relative frequencies of cancer cell types, in particular their asymptotic distribution, in populations seeded with samples from a heterogeneous population (Gupta et al. 2011; Pisco et al. 2013; Su et al. 2017; Risom et al. 2018; Dirkse et al. 2019; Najafi et al. 2023). This is empirical evidence that growth dynamics of cancer could be largely inconsequential for studies of its heterogeneity.

However, growth dynamics that are deemed realistic for the abundances of cancer cells often imply that the population eventually approaches a unique stable size. An example is logistic growth, under which the population size invariably stabilizes at carrying capacity. This invites a theoretical explanation for the observed irrelevance of population growth in studies of heterogeneity. Existence and uniqueness of an attracting stable state for growth dynamics suggest that transition dynamics among cell types may occur, by and large, around the equilibrium size, where growth dynamics tend to vanish and the distinction between abundance and relative frequency of cell types loses its significance. This argument, which is found for example in (Shah et al. 2023) on phenotypic heterogeneity, appears valid at an intuitive level. Yet, to the best of our knowledge, it has not been made formally rigorous so far.

Nonlinear dynamics in numerous systems are often crucial to our understanding of these systems. But, as we will show rigorously in the present manuscript, nonlinear dynamics of growth turn out to be ultimately irrelevant to modeling heterogeneity of cancer cells. We propose a generic modeling framework that combines nonlinear growth dynamics with linear transition dynamics for heterogeneous populations of cells. The framework can accommodate the main forms of nonlinear growth dynamics usually associated with cancer cell populations and is not committed to any specific kind, i.e. genetic or phenotypic, of heterogeneity. We then use the concept of asymptotic equivalence as defined by the qualitative theory of differential equations (Brauer and Nohel 1989) and related results (Yakubovich 1951; Brauer 1962). In particular, we prove that dynamics of the proposed model are asymptotically equivalent to those governed exclusively by the linear part of it. In the context of studying cancer heterogeneity, our work contributes to justify the use of linear models of cell-type transitioning that neglect cancer growth dynamics.

## Model

We model the dynamics of a cell population where $x_{i}$ is the abundance of cells of type *i* ($i=1,2,\dots ,n$) via the following differential equation

1 $$\begin{aligned} \dot{\varvec{x}}=\underbrace{g(N(\varvec{x}))\varvec{R}(t)\varvec{x}}_{\text {nonlinear part}}\quad +\underbrace{\varvec{A}\varvec{x}}_{\text {linear part}},\qquad \varvec{x}(0)=\varvec{x}_{0}, \end{aligned}$$

where $\dot{\varvec{x}}=\frac{\text {d}\varvec{x}}{\text {d}t}$, $\varvec{x}=[x_{1},x_{2},\dots ,x_{n}]^{\top }$, $\top$ indicates transposition, $\varvec{x}_{0}\ne 0$ is the initial population, which is a nonzero vector with non-negative components, while

2 $$\begin{aligned} N(\varvec{x})=\varvec{1}^{\top }\varvec{x}=\sum _{j=1}^{n}x_{j}, \end{aligned}$$

with $\varvec{1}=[1,1,\dots ,1]^{\top }$, is the total population size with $N_{0}=N(\varvec{x}_{0})>0$. In what follows, we will often use the streamlined notation *N* for $N(\varvec{x})$. The scalar-valued function *g*(*N*) is differentiable on $N>0$ and there exist positive constants $N_{1}$ and $N_{2}$ (with $N_{1}<N_{2}$), which may depend on the specific form of *g* and not be necessarily unique, such that

3 $$\begin{aligned} g(N)>0,\qquad 0<N<N_{1}, \end{aligned}$$

and

4 $$\begin{aligned} g(N)<0,\qquad N>N_{2}. \end{aligned}$$

With this formalism, we can introduce, for example, logistic growth by setting $g(N)=1-\frac{N}{K}$ or Gompertz growth by setting $g(N)=\ln \frac{K}{N}$ with *K* a positive constant and $N_{1}=\frac{K}{2}$ and $N_{2}=2K$.

The function *g* modulates $\varvec{R}(t)$, which is a diagonal matrix with (*i*, *i*)-entry ($i=1,2,\dots ,n$) equal to $r_{i}(t)\ge 0$. We take $r_{i}(t)$ to be equal to $b_{i}(t)-d_{i}(t)$, where $b_{i}(t)$ is the per-capita birth rate and $d_{i}(t)$ is the per-capita death rate at time *t*, so that $r_{i}(t)$ is the (possibly time-dependent) intrinsic growth of cells of type *i*. The $r_{i}(t)$ are uniformly bounded above by $r_{\max }$, i.e., $r_{\max }\ge |\varvec{R}(t)|$ for all $t\ge 0$, where we use the 1-norm for matrices, i.e. $|\varvec{M}|=\max _{j}\sum _{i}|m_{i,j}|$ for a matrix $\varvec{M}$. We also assume that the average

5 $$\begin{aligned} \bar{r}(t)=\frac{\sum _{i=1}^{n}r_{i}(t)x_{i}(t)}{\sum _{i=1}^{n}x_{i}(t)}=\frac{\varvec{1}^{\top }\varvec{R}(t)\varvec{x}(t)}{\varvec{1}^{\top }\varvec{x}(t)}=\frac{\varvec{1}^{\top }\varvec{R}(t)\varvec{x}(t)}{N(t)}\ge 0, \end{aligned}$$

of the type-specific growth rates weighted by cell-type abundance is integrable and takes value 0 at most a finite number of times so that, eventually, $\bar{r}(t)$ remains positive. In particular, there is some $t_{r}\ge 0$ such that

6 $$\begin{aligned} \bar{r}(t)\ge r_{min}>0,\qquad t\ge t_{r}. \end{aligned}$$

**Fig. 1:**
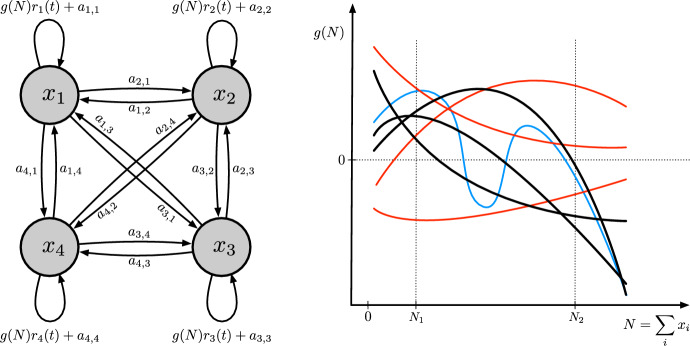
The left panel gives a schematic representation of the model in Eq. (1) for the case $n=4$. The right panel reports possible shapes of the function *g* in this model. Black lines represent functions that satisfy $g(N)>0$ for $0<N<N_{1}$ and $g(N)<0$ for $N>N_{2}$ for constants $N_{1}$ and $N_{2}$. These functions are within the scope of our model. Red lines represent functions that fail to satisfy these inequalities and are, therefore, out of the scope of the model. The blue line represents a function that is within the model scope, but since this function has more than one root the main result in this paper does not apply to this specific choice of the function *g*, see Sect. 4. For ease of visualization, in this panel the same $N_{1}$ and $N_{2}$ are kept for all depicted functions. However, these constants generally depend on the specific function *g* and, for a given function, they may not be unique

The linear part of Eq. (1) captures cell transitions among types and, ultimately, heterogeneity in the population. $\varvec{A}=[a_{i,j}]$ is a constant square matrix. Its non-diagonal entries in column *i* ($i=1,2,\dots ,n$) are non-negative and contain the transition rates from type *i* into other types, while the non-diagonal entries in row *i* ($i=1,2,\dots ,n$) of $\varvec{A}$ contain the transition rates from other types into type *i*. We impose the condition

7 $$\begin{aligned} \varvec{1}^{\top }\varvec{A}=\varvec{0}^{\top }, \end{aligned}$$

where $\varvec{0}=[0,0,\dots ,0]^{\top }$, so that cell flow out of each type equals cell flow into that type:

8 $$\begin{aligned} a_{j,j}=-\sum _{i\ne j}a_{i,j}. \end{aligned}$$

Fig. 1 gives a schematic representation of the model for the case $n=4$ and possible shapes for the function *g*.

Solutions of Eq. (1) are nonnegative and bounded. As for non-negativity, if $x_{i}(t)=0$, then $g(N)r_{i}(t)x_{i}(t)=0$ and the fact that non-diagonal entries of $\varvec{A}$ are non-negative imply that

9 $$\begin{aligned} \dot{x}_{i}=g(N)r_{i}(t)x_{i}+\sum _{j\ne i}^{n} a_{i,j} x_{j} =\sum _{j\ne i}^{n} a_{i,j} x_{j}\ge 0. \end{aligned}$$

As for boundedness, we note that, since for some constants $N_{1}$ and $N_{2}$ we have $g(N)>0$ for $0<N<N_{1}$ and $g(N)<0$ for $N>N_{2}$, then we have that $g(N)>0$ for $0<N<N_{1}/2$ and $g(N)<0$ for $N>2N_{2}$. Therefore, we can define $N_{1}'=N_{1}/2$, $N_{2}'=2N_{2}$, $U_{1}=\min \{N_{1}',N_{0}\}>0$ and $U_{2}=\max \{N_{2}',N_{0}\}$ and consider the compact set

10 $$\begin{aligned} H=\{\varvec{y}\in \mathbb {R}^{n}_{\ge 0}: U_{1}\le |\varvec{y}|\le U_{2} \}, \end{aligned}$$

where $\mathbb {R}^{n}_{\ge 0}$ is the non-negative orthant and $|\varvec{y}|$ is the 1-norm for vectors, i.e. $|\varvec{y}|=\sum _{i=1}^{n}|y_{i}|$. Taking the time derivative of Eq. (2) while using Eqs. (1) and (7),

11 $$\begin{aligned} \dot{N}=\varvec{1}^{\top }\dot{\varvec{x}} =g(N)\varvec{1}^{\top }\varvec{R}(t)\varvec{x}+\varvec{1}^{\top }\varvec{A}\varvec{x}=g(N)\varvec{1}^{\top }\varvec{R}(t)\varvec{x}. \end{aligned}$$

Since $\varvec{1}^{\top }\varvec{R}(t)\varvec{x}\ge 0$ with equality holding when $\bar{r}(t)=0$, the sign of $\dot{N}$ is either 0, when $\bar{r}(t)=0$, or equal to the sign of *g*(*N*). When $N=U_{1}$, $0<N\le N_{1}'<N_{1}$ and, by Eqs. (3) and (11), $g(N)> 0$ and $\dot{N}\ge 0$, while when $N=U_{2}$, $N\ge N_{2}'> N_{2}$ and, by Eqs. (4) and (11), $g(N)< 0$ and $\dot{N}\le 0$. Therefore, *H* is positively invariant for Eq. (1). Since $N_{0}\in H$, all solutions of Eq. (1) are found within *H* and, therefore, are bounded.

## Asymptotic Equivalence

Our main result consists in showing that, under certain conditions, the dynamics of our model are ultimately governed only by its linear part. To get to this result, we first need to introduce the concept of asymptotic equivalence between differential equations. Consider the following two systems of *n* equations

12a $$\begin{aligned} \dot{\varvec{x}}=\varvec{f}(t,\varvec{x})+\varvec{M}\varvec{x}, \end{aligned}$$

12b $$\begin{aligned} \dot{\varvec{y}}=\varvec{M}\varvec{y}, \end{aligned}$$

both defined on $t\ge 0$ where $\varvec{M}$ is a $n\times n$ matrix, $\varvec{x}$ and $\varvec{y}$ are vectors of *n* components and $\varvec{f}$ is a continuous vector-valued function. Following (Brauer 1962, Section 2), we say that these two systems are asymptotically equivalent if for every solution $\varvec{x}(t)$ of Eq. (12a) there exists a solution $\varvec{y}(t)$ of Eq. (12b) such that

13 $$\begin{aligned} \lim _{t\rightarrow \infty }|\varvec{x}(t)-\varvec{y}(t)|= 0, \end{aligned}$$

and, conversely, for every solution $\varvec{y}(t)$ of Eq. (12b) there exists a solution $\varvec{x}(t)$ of Eq. (12a) such that Eq. (13) holds.

To establish asymptotic equivalence between Eq. (12a) and Eq. (12b), we will use a result from (Yakubovich 1951, Theorem 2), which we present without proof in the form found in (Brauer 1962, Theorem 6),

### Theorem 1

(Yakubovich) Let $\mu$ be the largest among the real parts of the eigenvalues of $\varvec{M}$. Let *m* be the largest algebraic multiplicity of the eigenvalues of $\varvec{M}$ that have real part $\mu$. Let *q* be the largest algebraic multiplicity of any eigenvalue of $\varvec{M}$ with real part equal to 0 with $q=1$ in case no eigenvalue of $\varvec{M}$ has real part equal to 0. If there is a function $\eta (t)$ such that $|\varvec{f}(t,\varvec{x})|\le \eta (t)|\varvec{x}|$ and

14 $$\begin{aligned} \int _{0}^{\infty }t^{m+q-2}e^{t\mu }\eta (t)\text {d}t <\infty , \end{aligned}$$

then Eq. (12a) and Eq. (12b) are asymptotically equivalent.

When Eq. (12a) and Eq. (12b) are asymptotically equivalent, the former system, which is possibly nonlinear, can be seen as a perturbed version of the latter, linear system where the perturbation becomes eventually irrelevant. The notion of asymptotic equivalence is particularly useful when stability properties of a nonlinear system are difficult to obtain, yet it can be shown that the system asymptotically behaves as a linear system that is hopefully easier to analyze (Brauer 1962, Section 4.1). Asymptotic equivalence means that one can always find solutions of the two systems such that $|\varvec{x}(t)-\varvec{y}(t)|$ is *o*(1). However, this notion is limited in that it does not directly yield quantitative estimates of the error term $|\varvec{x}(t)-\varvec{y}(t)|$ and of how fast this goes to 0. Explicit estimates for the difference between the fundamental matrices of two asymptotically equivalent systems in the special case in which $\varvec{f}(t,\varvec{x})$ is linear are derived in (Bodine and Lutz 2010).

## Main Result

We consider the following two systems:

15a $$\begin{aligned} \dot{\varvec{x}}=g(N)\varvec{R}(t)\varvec{x}+\varvec{A}\varvec{x}, \end{aligned}$$

15b $$\begin{aligned} \dot{\varvec{y}}=\varvec{A}\varvec{y}, \end{aligned}$$

defined on $t\ge 0$, where Eq. (15a) is our model in Eq. (1) and Eq. (15b) is a system corresponding to the sole linear part of our model. Our aim is to provide a sufficient condition for the asymptotic equivalence between Eq. (15a) and Eq. (15b). To this aim, we first establish the following preliminary result:

### Proposition 1

$\varvec{A}$ has an eigenvalue equal to 0 and no eigenvalue of $\varvec{A}$ has real part larger than 0.

### Proof

Eq. (7) shows that 0 is an eigenvalue of $\varvec{A}$ with corresponding left eigenvector $\varvec{1}$. By Gers̆gorin theorem (Horn and Johnson 2013, Corollary 6.1.3), all eigenvalues of $\varvec{A}$ are in the union of the closed disks centered at $a_{j,j}$ ($j=1,2,\dots ,n$) with radius $\sum _{i\ne j}|a_{i,j}|$. By assumption, every column of $\varvec{A}$ contains non-negative non-diagonal entries that add up to minus the diagonal entry in that column. Every Gers̆gorin’s disk of $\varvec{A}$ then has its center on the non-negative part of the real axis of the complex plane and all circles enclosing the disks pass through the origin. Hence, the real part of every eigenvalue of $\varvec{A}$ is at most 0. $\square$

We can now state our main result:

### Theorem 2

If there exists a function *h* of *N* that is positive and differentiable on $N>0$ and solves the following differential equation,

16 $$\begin{aligned} -\dfrac{C}{Nh}=\dfrac{\text {d}}{\text {d}N}\dfrac{g}{h},\qquad N>0, \end{aligned}$$

where *C* is a positive constant, then the system in Eq. (15a) and the system in Eq. (15b) are asymptotically equivalent.

### Proof

Suppose *h* exists. Let

17 $$\begin{aligned} V(N)=\frac{\sqrt{g(N)^{2}}}{h(N)}, \end{aligned}$$

which is positive on $N>0$. Differentiating this equation with respect to time while using Eqs. (2), (5) and (11),

18 $$\begin{aligned} \dot{V} =\dfrac{\dot{N}g(N)}{\sqrt{g(N)^{2}}}\frac{\text {d}}{\text {d}N}\frac{g(N)}{h(N)} =-\dfrac{CV}{N}\varvec{1}^{\top }\varvec{R}\varvec{x} =-CV\bar{r}, \end{aligned}$$

Solving this differential equation and then using Eq. (6), we obtain a differential inequality,

19 $$\begin{aligned} \begin{aligned} V(t)&= V(0) \exp \left( -C\int _{0}^{t} \bar{r}(s)\text {d}s\right) \\&\le V(0) \exp \left( -C\int _{0}^{t_{r}}0\text {d}s-C\int _{t_{r}}^{t}r_{\min }\text {d}s\right) \\&=V(0)e^{Cr_{\min }t_{r}} e^{-Cr_{\min }t}. \end{aligned} \end{aligned}$$

The dynamics of *N* in Eq. (15a) are bounded with $0<U_{1}\le N\le U_{2}<\infty$ for all $t\ge 0$, see Eq. (10). By the extreme value theorem, the continuous functions *h*(*N*) and *V*(*N*) achieve maximal values $Q_{1}>0$ and $Q_{2}>0$, respectively, on $[U_{1},U_{2}]$. With these bounds, the inequality in Eq. (19) and the definition of *V* we can bound the nonlinear part of Eq. (15a),

20 $$\begin{aligned} \begin{aligned} |g(N(t))\varvec{R}(t)\varvec{x}|&\le r_{\max }|g(N)||\varvec{x}|\\&=r_{\max }h(N(t))V(N(t))|\varvec{x}|\\&\le r_{\max } Q_{1}V(N(t))|\varvec{x}|\\&\le r_{\max }Q_{1}e^{Cr_{\min }t_{r}}e^{-tCr_{\min }} V(N(0))|\varvec{x}|\\&\le r_{\max }Q_{1}Q_{2}e^{Cr_{\min }t_{r}}e^{-tCr_{\min }}|\varvec{x}|. \end{aligned} \end{aligned}$$

We can now invoke Theorem 1 and the notation therein. Let $\varvec{f}(t,\varvec{x})=g(N(t))\varvec{R}(t)\varvec{x}$, $\varvec{M}=\varvec{A}$ and $\eta (t)=r_{\max }Q_{1}Q_{2}e^{Cr_{\min }t_{r}}e^{-tCr_{\min }}$ so that, by Eq. (20), $|\varvec{f}(t,\varvec{x})|\le \eta (t)|\varvec{x}|$. By Proposition (1), we have $\mu =0$, $1\le m\le n$ and $1\le q\le n$. Then, $p=m+q-2$ is a non-negative integer with maximum value $2(n-1)$. Substituting in Eq. (14), the improper integral therein converges

21 $$\begin{aligned} \begin{aligned} \int _{0}^{\infty }t^{p}\eta (t)\text {d}t&=r_{\max }Q_{1}Q_{2}e^{Cr_{\min }t_{r}}\int _{0}^{\infty }t^{p}e^{-tCr_{\min }}\text {d}t\\&=r_{\max }Q_{1}Q_{2}e^{Cr_{\min }t_{r}}(Cr_{\min })^{-p-1}\Gamma (p+1)\\&=p!r_{\max }Q_{1}Q_{2}e^{Cr_{\min }t_{r}}(Cr_{\min })^{-p-1}, \end{aligned} \end{aligned}$$

where $\Gamma$ is the gamma function and the solution of the integral on the right-hand side of the first line of this equation is found in (Gradshteyn et al. 2014, 2.325 6$^{*}$). Therefore, the system in Eq. (15a) and the system in Eq. (15b) are asymptotically equivalent. $\square$

A corollary of Theorem 2 is that there is a unique, stable equilibrium of the dynamics of *N* on $[U_{1},U_{2}]$. Since $g(U_{1})>0$ and $g(U_{2})<0$, the function *g* has at least one root $N^{*}>0$ in the interior of this interval so that $g(N^{*})=0$. If *h* exists as specified in Theorem 2, then Eq. (16) at $N^{*}$ reduces to $-C=N^{*}g'(N^{*})$. Since both *C* and $N^{*}$ are positive, $g'(N^{*})$ is negative for every root $N^{*}$ of *g*. But continuity of *g* implies that there cannot be more than one such root. This proves the corollary. It can also be shown that $N^{*}$ is attracting on $[U_{1},U_{2}]$ because, as Eqs. (18-20) show, $V\rightarrow 0$ as $t\rightarrow \infty$, and, by Eq. (17), $V=0$ if and only if $g(N)=0$, but $g(N)=0$ if and only if $N=N^{*}$. This corollary reveals a limitation of Theorem 2: it does not apply when *g* in our model in Eq. (1) has more than one root (Fig. 1).

A further corollary is the existence of a carrying simplex $\Sigma$ defined by the set of population state vectors with 1-norm equal to $N^{*}$. If the function *h* exists as specified in Theorem 2, then on the simplex $\Sigma$ the nonlinear part of the system in Eq. (1) vanishes (because the function *g* does) and only the linear system in Eq. (15b) is left. The simplex $\Sigma$ is positively invariant for this linear system because its dynamics preserve the 1-norm: by Eq. (7), $\frac{\text {d}}{\text {d} t}|\varvec{y}|=\varvec{1}^{\top }\dot{\varvec{y}}=\varvec{1}^{\top }\varvec{Ay}=0$.

## Application to Nonlinear Dynamics of Cancer Growth

Table 1 shows that Theorem 2, despite the limitations stated in the previous section, is general enough to apply to the three main forms of nonlinear dynamics that are usually believed to govern the growth of cancer cells populations (Spratt et al. 1993; Gerlee 2013; Benzekry et al. 2014; Hartung et al. 2014; Sarapata and Pillis 2014; Diebner et al. 2018; Vaghi et al. 2020): generalized logistic growth, generalized Gompertz growth and generalized von Bertalanffy growth. The corollary to this theorem ensures that the cancer cell population will approach the equilibrium size $N^{*}$ with $N^{*}=K$ for generalized logistic and Gompertz growth and $N^{*}=(w/v)^{-\frac{1}{\gamma }}$ for generalized von Bertalanffy growth.

**Table 1:** Common nonlinear growth dynamics for cancer cell populations accommodated by the proposed model

Growth dynamics	*g*(*N*)	*h*(*N*)	*C*
Generalized logistic growth	$1-\left( \dfrac{N(\varvec{x})}{K}\right) ^{\gamma }$	$N(\varvec{x})^{\gamma }$	$\gamma$
Generalized Gompertz growth	$\ln \left[ \left( \dfrac{K}{N(\varvec{x})}\right) ^{\gamma }\right]$	1	$\gamma$
Generalized von Bertalanffy growth	$vN(\varvec{x})^{-\gamma }-w$	$N(\varvec{x})^{-\gamma }$	$w\gamma$

*K*, $\gamma$, *v* and *w* are positive parameters. Within each row, the functions *g* and *h* along with the constant *C* solve the differential Eq. (16)

## Discussion

A corollary of our main result (Theorem 2) is the existence of a carrying simplex, where only the linear dynamics of the model in Eq. (1) persist while the nonlinear dynamics vanish. This suggests two separate considerations:

The first consideration has to do with time-scale separation. We may hypothesize that the nonlinear part of our system vanishes faster than the linear part as the carrying simplex is approached so that the system quickly reduces to its linear part. While working with multiple time scales is an effective approach in the analysis of differential equations (Kuehn 2015), a strength of our results is their independence of the existence of a time-scale separation between the nonlinear part and the linear part of Eq. (1). To understand why, suppose we replace $\varvec{A}$ with $\epsilon _{1}\varvec{A}$, for some $\epsilon _{1}>0$, in the linear part of Eq. (1). Changing the value of $\epsilon _{1}$ we can arbitrarily vary the speed of the dynamics of the linear part of the system. Yet, Theorem 2 still holds. This is because if Eqs. (7) and (8) hold for $\varvec{A}$, then equivalent equations hold for $\epsilon _{1}\varvec{A}$ and if Proposition 1 holds $\varvec{A}$, then an equivalent proposition holds for $\epsilon _{1}\varvec{A}$ because the spectrum of the latter matrix is equal to the spectrum of the former scaled by $\epsilon _{1}$. We could also independently alter the speed of the nonlinear part of Eq. (15a). After placing an arbitrary positive factor $\epsilon _{2}$ in front of $g(N)\varvec{R}(t)\varvec{x}$ in Eq. (15a), Theorem 2 would still hold with the sole modification that $\epsilon _{2}$ would now appear as a factor on both sides of Eq. (20). But this would not affect the convergence of the integral in Eq. (21). Our main finding in Theorem 2 is then compatible with an intrinsic time-scale separation between the dynamics of the nonlinear part and those of the linear part of the system in Eq. (1). Yet the result is general enough to lead to a positive conclusion about the asymptotic equivalence of our system to its linear part independently of the existence of a time-scale separation and, in case of existence of this separation, independently of which part of the system is faster.

However, asymptotic equivalence is not, in general, independent of how fast the dynamics of the nonlinear part of a system are compared to those of its linear part. In our case, independence derives from the fact that scaling of $\varvec{A}$ by a positive factor does not alter the relevant properties of this matrix, in particular those of its spectrum, for Theorem 2 to apply. But looking at the more general Theorem 1 about asymptotic equivalence, if $\varvec{M}$ has positive dominant eigenvalue $\mu$, then scaling of this matrix by $\epsilon >0$ implies scaling of this eigenvalue by the same factor. This, in turn, sets conditions (dictated by the term $e^{t\epsilon \mu }$) on how fast the nonlinear part of Eq. (12a) should vanish so that the integral in Eq. (14) converges and one can establish asymptotic equivalence between Eqs. (12a) and (12b).

The second consideration relates to the work of Smale (Smale 1976), who proved that an arbitrary dynamical system in $n-1$ dimensions can be embedded in a competitive system of *n* species the asymptotic dynamics of which are restricted to a $(n{-}1)$-dimensional carrying simplex. Species dynamics on this carrying simplex would then be fully governed by the embedded system. By identifying species as cell types, Smale’s result suggests an approach alternative to ours to combine growth dynamics of, and transition dynamics within, cell populations that also allows one to ultimately neglect growth dynamics and exclusively focus on transition dynamics. Only looking at the nonlinear part of Eq. (1), growth dynamics as specified in Table 1 can in fact be employed to define a competitive system with a carrying simplex. Transition dynamics of cells could then be embedded in the competitive system as opposed to added to it as in our Eq. (1).

However, Smale’s construction requires the dynamics of the embedded system to be “small", for otherwise the system may fail to be competitive. An advantage of our framework is that this restriction is not required. On the other hand, an approach based on Smale’s construction can be more flexible than ours in accommodating nonlinear aspects of cancer dynamics other than cell-type specific growth rates. In modeling cancer cell populations, it is common to postulate interactions among cells. Lotka-Volterra equations (Hofbauer and Sigmund 1998, Section 5.1) define a nonlinear system that has been shown to aptly capture interactions between healthy and cancerous cells (Foryś 2009; Grigorenko et al. 2020) and between cancer cells (Freischel et al. 2021; Cho et al. 2023; Gérard et al. 2024). Competitive Lotka-Volterra equations with a carrying simplex easily lend themselves to (and, in fact, were an inspiration for) Smale’s construction. It is unclear to us, instead, how interactions of the Lotka-Volterra type could be included in the nonlinear part of an equation like Eq. (12a) while ensuring asymptotic equivalence of this to its linear part.

Finally, we would like to recall the motivating factor behind our work: the success of linearity assumptions in modeling experimental data of transitions among cancer cell types (Gupta et al. 2011; Pisco et al. 2013; Su et al. 2017; Risom et al. 2018; Dirkse et al. 2019; Najafi et al. 2023) in the face of nonlinearities inherent to the growth dynamics of cancer. We believe that our results, despite their stated limitations, suggest a possible explanation for this success.

## Data Availability

There are no data or code associated with this manuscript.
